# Improved Cell Allocation Strategies Using K-Means Clustering in Congested 6TiSCH Environments

**DOI:** 10.3390/s24175608

**Published:** 2024-08-29

**Authors:** Fransiskus Xaverius Kevin Koesnadi, Sang-Hwa Chung

**Affiliations:** Department of Information Convergence Engineering, Pusan National University, Busan 46241, Republic of Korea; fxkevink@pusan.ac.kr

**Keywords:** 6TiSCH, cell allocation, K-means, node density, wireless sensor network

## Abstract

The 6TiSCH protocol (IEEE 802.15.4e) is crucial for the Industrial Internet of Things (IIoT), utilizing a time-slotted channel hopping (TSCH) mode based on node distribution. In this study, we propose an innovative cell allocation strategy based on node position clustering using the K-means algorithm, specifically designed to address congestion and optimize resource distribution in the 6TiSCH network. Our mechanism effectively groups nodes into clusters, allowing for dynamic adjustment of cell capacities in congested areas by analyzing traffic patterns and the spatial distribution of nodes. This clustering approach enhances the efficiency of slot frame utilization and minimizes communication delays by reducing interference and improving routing stability. The proposed strategy leverages the clustering results to improve cell usage efficiency and reduce communication latency between nodes. By tailoring cell allocation to the specific traffic needs of each cluster, we significantly reduce packet loss, manage congestion more effectively, and enhance data transmission reliability. We evaluated the clustering method using the K-means algorithm through experiments with the 6TiSCH simulator. Additionally, we considered using objective functions in Routing Protocol for Low-Power and Lossy Networks (RPL), such as OF0 and MRHOF, to assess clustering results and their impact on throughput and packet delivery. Our method resulted in significantly improved average performance metrics. Under the OF0 routing protocol, we achieved a 30.01% latency reduction, a 15.95% faster joining time, an 8% higher packet delivery ratio, and a 13.82% throughput increase. Similarly, we observed a 12.34% improvement in packet delivery ratio, 21.06% latency reduction, 12.68% faster joining time, and 25.97% higher throughput speed with the MRHOF routing protocol. These findings highlight the effectiveness of the improved cell allocation strategy in congested 6TiSCH environments, offering a better solution for enhancing network performance in IIoT applications.

## 1. Introduction

The Internet of Things (IoT) connects physical devices, enabling wireless sensor nodes to share environmental data and facilitate system interaction with the real world. Wireless sensor networks (WSNs) have gained interest for their diverse applications in analyzing environmental conditions and guiding appropriate responses [1]. 6TiSCH addresses the needs of low-power wireless networks by ensuring end-to-end reliability and deterministic latency. It integrates IPv6 with IEEE802.15.4 time-slotted channel-hopping (TSCH), providing industrial-grade performance and seamless internet connectivity that are vital for the Industrial Internet of Things (IIoT) [2]. Therefore, the present work identifies and addresses the challenges in routing strategies and security management to enhance their efficiency and effectiveness while striving for greater simplicity in these areas [3,4]. In IEEE 802.15.4’s TSCH mode, limited broadcast opportunities occur at specific times and channels. Routers use enhanced beacon (EB) frames to announce the network; these EBs can include critical information that allows new or long-sleeping nodes to make the most of these rare broadcasts, thereby ensuring efficient slot usage [5]. Scheduling techniques in TSCH networks vary, with some using centralized methods employing root node managing schedules and cell allocations and others using link-based schemes employing MAC addresses. However, current TSCH approaches may not meet future IIoT needs. Hence, more lightweight and energy-efficient scheduling and routing algorithms are necessary [6].

The 6TiSCH architecture is crucial for the IIoT, ensuring reliable communication through effective cell allocation to prevent packet loss in high-traffic areas. In factories, field devices and sensors require high reliability and timely information exchange [7]. Efficient cell allocation based on node position directly influences network performance and power consumption [8]. Optimizing cell allocation in 6TiSCH-based wireless sensor networks is essential for reliable data transmission, especially in densely deployed environments [9] comprising industrial automation, smart city infrastructure, and environmental monitoring implementations. This work employs the K-means algorithm for label-free clustering to optimize cell allocation in 6TiSCH networks by clustering nodes based on their spatial distribution and link performance, which helps to avoid the need for further iterations. Centroid nodes are designated as cluster heads and other nodes are associated with the nearest center, resulting in reduced transmission hops, simplified neighbor discovery, and enhanced scalability. This clustering strategy informs a dynamic cell allocation approach within the slot frame, which improves communication efficiency and minimizes collisions. Our proposed method efficiently manages intra-cluster subchannel allocation and inter-cluster interference through distributed learning, which optimizes cell utilization, reduces overhead, and enhances overall network efficiency. This optimization is reflected in the network topology, where parent nodes function as cluster heads and sink nodes serve as data collection centers. Dynamic cell allocation within each cluster is guided by communication demands and traffic density, thereby minimizing congestion and improving transmission efficiency. Furthermore, routing paths and network organization are determined by RPL while considering factors such as OF0 and MRHOF. Network performance is assessed based on the joining time, packet delivery ratio, end-to-end latency, and throughput. By maintaining a constant number of nodes, this study prioritizes the evaluation of clustering and cell allocation strategies while accounting for the static conditions typical of industrial and IIoT environments [10].

Our goal is to enhance resource utilization in 6TiSCH networks and to reduce packet loss based on the node distribution. K-means clustering is applied to nodes based on nearby communication needs to optimize packet routes, minimize packet loss and latency, and ensure reliable data communication by division based on nodes’ distance in a grid area. This method also prevents slot collisions and maintains efficient cell placement by ensuring that cells from the same communication node do not occupy the same slot frame. The proposed system allocates additional slots in the next available frame if the available slots are insufficient.

The contribution of this study can be summarized as follows:

- We improve cell allocation in the 6TiSCH network, optimizing resource utilization, reducing packet loss, and ensuring reliable communication.
- We utilize K-means clustering to group nodes based on nearby communication needs, which minimizes packet loss and latency, improves delivery ratio and throughput, and prevents slot collisions by maintaining efficient cell placement.
- We enhance network performance and scalability for IIoT applications by providing insights into node distribution, communication patterns, and resource allocation, enabling better management of network resources and avoiding slot clashes through dynamic cell allocation.

The structure of this paper is as follows: Section 2 provides an overview of the simulator and tools, and discusses the problem of clustering techniques; Section 3 reviews related works for comparison; Section 4 outlines the proposed density clustering mechanism for node-centric cell allocation in the 6TiSCH network; Section 5 presents the performance evaluation, covering metrics such as packet delivery ratio, latency, time allocation for every node to join the network, and throughput; finally, Section 6 concludes the paper with a summary of the findings.

## 2. Related Work

This section provides an overview of studies on clustering techniques in wireless sensor networks and related research. Our method employs group nodes for administration and distributed job execution, including management of resources such as the congestion level of neighboring nodes. Although the primary benefit of clustering systems is lower energy consumption, clustering also addresses network heterogeneity and mobility, load balancing, and energy conservation.

One application of cluster formation in WSN, as demonstrated by [11], employs particle swarm optimization and Tabu algorithm techniques. These methods improve energy efficiency, network longevity, and protocol performance by rotating cluster head roles based on energy levels, utilizing multi-hop routing algorithms, and incorporating fault tolerance mechanisms. However, the selection of cluster heads remains constrained by the parameter settings, which can result in elevated energy consumption. Space partition in K-means clustering using optimal K-means was employed in [12] to address area partition by forming space partitions in a wireless sensor network, although the node issuance algorithm remains unclear. A modified grid-based wireless sensor network utilizing the K-means approach [13] has been tested to enhance energy efficiency and extend the lifespan of large-scale wireless sensor networks. This method reduces overall energy consumption and improves network longevity by dividing the network area into grids and applying K-means clustering within each grid cell; however, it may impose limitations on deploying heterogeneous sensor networks that rely on a static parent node.

Hierarchical protocols such as Low-Energy Adaptive Clustering Hierarchy (LEACH) reduce the amount of data transmitted across the network by dividing it into clusters with cluster heads responsible for data aggregation and relaying. This protocol conserves energy and improves scalability. Modifying LEACH, as proposed by [14], combines the midpoint algorithm with the K-means approach to enhance network lifespan in wireless sensor networks, aiming to create balanced clusters and reduce cluster head load. Further optimization of this method could improve overall network efficiency and prolong network lifespan, addressing the minor declines in the packet delivery ratio observed in initial tests. As mentioned earlier, LEACH can be adapted to anticipate reduced data transmission, as suggested by [15], where an algorithm was developed to select a primary and secondary cluster head in order to maintain network performance and efficiency while ensuring data flow continuity even in the event of node failure. Another method [16] focuses on improving the average energy consumption per node in each round and the number of surviving nodes per round, although routing algorithms still need to address high latency issues.

To minimize frequent reclustering and associated energy overhead, [17] introduced a multi-hop routing algorithm to optimize energy use across the network, incorporating a fault-tolerant mechanism to address CH and relay node failures. However, this experiment’s routing process emphasized close transmission distances without considering the need for nodes to be utilized, aiming to minimize energy consumption even if the receiver node remains inactive without any expected packet reception. In [18], the authors employed a joint optimization strategy using the A3C learning algorithm to minimize the system energy consumption of the NOMA cluster while ensuring safe and low-delay computational loading in dynamic vehicle networks.

Our research focuses on cell allocation and its impact on wireless sensor network implementation, unlike previous works that typically concentrate on node localization. We examine how node grouping performance affects each cell allocation, particularly in TSCH, as well as its indirect relation to the cell allocation system in WSN, while considering node location, energy efficiency, and network performance. Unlike earlier studies, we investigate how group node positioning influences cell operation in TSCH, focusing on scalability, throughput delay, and reliable communication. Furthermore, we propose a dynamic allocation method for enhancing network efficiency that makes adjustments based on node mobility and network responsiveness during data transmission.

## 3. Background

### 3.1. TSCH

IEEE 802.15.4e Time-Slotted Channel Hopping (TSCH) is an amendment to the IEEE 802.15.4 standard outlining the physical (PHY) and medium access control (MAC) layers for low-rate wireless personal area networks (WPANs). Designed for industrial and automation applications, TSCH offers synchronized time-based communication, making it dependable and energy-efficient. It is ideal for applications that require precise timing and coordination, such as industrial control systems or environmental sensor networks [19]. While TSCH specifies functionality, its implementation depends on network architecture and application needs. Channels are managed by dividing time into fixed time slots and periodically changing frequency channels, with each node assigned specific times for data transmission or reception and synchronized with neighboring nodes, thereby minimizing channel access conflicts [20].

TSCH is a MAC mode in the IEEE 802.15.4e standard that allocates time slots for data transmission or reception. These allocations can be static or dynamic based on network requirements. Time slots are organized into slot frames, which define cycles of time slots that repeat according to the slot frame size [21]. Nodes communicate within these time slots, where they are synchronized through a shared channel hopping schedule that periodically changes the frequency channels to reduce interference. This ensures precise communication and time synchronization between nodes, with the slot frame length being adjustable based on network and application needs. TSCH operation utilizes about 16 available channels, which allows for high coordination within the network and reduced channel access conflicts [22].

### 3.2. 6TiSCH Minimal Scheduling Function

6TiSCH (IPv6 over IEEE 802.15.4e TSCH mode) is an IETF standard for facilitating IPv6 communications over IEEE 802.15.4e TSCH mode [3]. It defines control plane protocols to align link-layer resources with application needs and integrates CoAP with link-layer security [23]. The 6TiSCH architecture combines IPv6 with IEEE 802.15.4e TSCH MAC, organizing time into slots within slot frames in which each slot is a distinct time interval for node functions [24,25]. Cells representing communications capability are controlled by the 6TiSCH Operational Sublayer Protocol (6P), which manages cell allocations and time slot usage to ensure reliable communication and resource efficiency in IIoT environments [25,26].

The Minimal Scheduling Function (MSF) in 6TiSCH networks adjusts cell allocation based on utilization metrics to meet dynamic bandwidth requirements [27]. Using TSCH at the MAC layer, MSF operates with minimal cells for network initialization, autonomous cells for default communication and negotiated cells for managing communication load [28]. MSF coordinates time slot and cell distributions, synchronizes node transmission schedules, and assigns specific roles to nodes [29]. This protocol ensures efficient bandwidth use, low latency, and high dependability, making it suitable for real-time control and monitoring systems [25].

### 3.3. Routing Protocol for Low-Power and Lossy Networks (RPL)

The Routing Protocol for Low-Power and Lossy Networks (RPL) is designed for resource-constrained environments such as wireless sensor network. RPL offers efficient and reliable routing by addressing challenges such as node mobility, dynamic topologies, and unpredictable conditions. It supports hierarchical structures and uses the Destination-Oriented Directed Acyclic Graph (DODAG) to establish optimal paths toward specific destinations while considering topology and delivery needs [30]. RPL also dynamically reorganizes paths in response to network changes, making it ideal for WSN and IoT applications thanks to its efficiency, reliability, and ability to manage control overhead and memory constraints [31].

#### 3.3.1. Objective Function Zero (OF0)

The Objective Function Zero (OF0) in the RPL guides nodes in selecting and optimizing routes within an RPL instance by using available information objects. Instead of providing specific instructions, OF0 offers a general concept to calculate a node’s rank by adding a normalized scalar to the rank of its preferred parent $R_{p}$. OF0 encodes rank in units of 256, allowing for hop ranges from 28 (worst) to 255 (best). In RPL, all parents are feasible successors for upward traffic, and nodes can consider parents in subsequent DODAG versions as potential successors. To compute the current node’s rank $R_{N}$, the step of rank $S_{p}$ is multiplied by the rank factor $R_{f}$ and adjusted with a stretch factor $S_{r}$ that is constrained by the configured rank stretch. This rank is then added to the preferred parent’s rank to determine the node’s overall rank. By calculating the rank, OF0 enables a node to participate in a DODAG version that provides satisfactory connectivity, with the minimal hop rank increase defining the lowest rank increase to any potential parent. OF0 does not guarantee optimization according to any specific metric; connectivity validation is specific to implementation and link type, which falls outside OF0’s scope [32].

#### 3.3.2. Minimum Rank with Hysteresis Objective Function (MRHOF)

Another RPL protocol, Minimum Rank with Hysteresis Objective Function (MRHOF), looks for the best path between source and destination nodes in resource-constrained wireless networks with changeable conditions. MRHOF aims to minimize routing overhead while balancing path quality and network stability. MRHOF evaluates each node’s rank value to find the shortest path using the minimum rank algorithm. MRHOF uses hysteresis to prevent frequent changes and preserve path stability in the face of varying network conditions. Additive metrics are compatible with this protocol because they use metrics to minimize path costs [33].

### 3.4. Unsupervised Learning and K-Means Clustering

Unsupervised learning aims to uncover patterns in unlabeled data by discovering hidden structures without predefined labels. Unlike supervised learning, which uses labeled datasets, unsupervised learning focuses on clustering, identifying correlations, and reducing data dimensionality. The clustering process relies on similarity metrics, with the Euclidean distance being the most common [34]. Other metrics, such as the correlation coefficient, may be used as well. Cluster analysis identifies high-density groups with similar observations within clusters. The number of clusters and similarity metric both have significant impacts on the results [35]. The K-means algorithm is a widely used unsupervised technique for clustering data into predefined clusters [36]. It partitions a dataset of *N* observations into *k* clusters by iteratively assigning observations to the nearest centroid, calculated as the mean of the points in a cluster. Starting with random centroids, the algorithm alternates between assigning observations based on the Euclidean distance and recalculating the centroids until reaching either convergence or a set number of iterations [37]. Using the Euclidean distance, K-means clustering seeks to minimize the within-cluster sum of squares (WCSS). Challenges include handling non-spherical clusters, outliers, and empty clusters, which are often resolved by reassignment or adjusting the number of clusters *k*. The elbow method determines the optimal *k* by plotting the WCSS against various values of *k* to find the ‘elbow’ point. Silhouette analysis assesses cluster compactness and separation, aiding in the selection of an appropriate *k* value.

#### Silhouette Score and Elbow Method

The Silhouette Score measures how well an object fits within its cluster compared to other clusters, ranging from −1 to 1. Positive values indicate that objects are more similar to their cluster members than those in other clusters, while negative values suggest the opposite [38]. For an object *i*, the cluster *B* with the minimum distance to *i* (i.e., d(i,B)=b(i)) is considered the closest alternative if *i* cannot be assigned to cluster *A*. Determining b(i) requires considering clusters other than *A*; hence, *k* must be greater than 1. The Silhouette Score s(i) combines a(i) and b(i) to evaluate cluster quality [38].

In K-means clustering, the elbow method identifies the optimal number of clusters *k* by plotting the distortion (inertia value) against *k*. The distortion measures the total distance between points and their cluster center. As *k* increases, points become closer to their centers, reducing the distortion. The rate of decrease slows, forming an elbow in the plot. The *k* at this elbow is optimal, as further increases in *k* yield minimal improvements in the explained variance [39].

## 4. Node Clustering

### 4.1. Cluster Initialization and Parameter Enhancement

**Figure 1:**
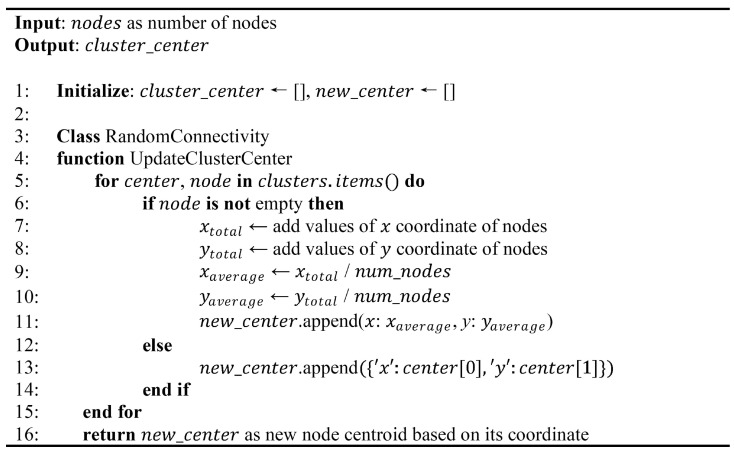
Pseudocode for K-means cluster center initialization and updating.

Initially, cluster centers are determined based on their coordinates in the random mote connectivity matrix. The central node of each cluster $C_{i}$ randomly initializes the centers within the data space, with the boundaries defined by the *k* values. A random integer generator within a specified range, represented by the coordinates ($x_{N}$, $y_{N}$), determines each cluster center. The algorithm in Figure 1 details the initialization and update procedures for the K-means cluster center. The process starts by randomly selecting the first cluster center from the given range for each coordinate (*x*, *y*). The mechanism then iteratively updates the cluster center for each cluster using the average coordinates of the nodes within that cluster. This process continues until convergence, ensuring that the cluster center stabilizes and does not change significantly with subsequent iterations, achieving a new_center of the coordinate.

### 4.2. Clustering Parameter Enhancement

The coordinates of the cluster centers ($x_{C_{i}}^{\prime }$, $y_{C_{i}}^{\prime }$) are iteratively updated using the K-means optimization method based on the average coordinates of the nodes within each cluster $C_{i}$. To assign nodes to their nearest clusters, the algorithm computes the Euclidean distance $d(N,C_{i})$ between each node *N* and the updated cluster centers ($x_{C_{i}}^{\prime }$, $y_{C_{i}}^{\prime }$) as shown in Equation (1), starting from the previous centers ($x_{C_{i}}$, $y_{C_{i}}$). This process establishes proximity-based clusters with each node’s coordinates denoted by $x_{N}$ and $y_{N}$, contributing to the calculation of the updated cluster centers.

(1) $$d\left(N,C_{i}\right)=\sqrt{{\left(x_{N}-x_{C_{i}}\right)}^{2}+{\left(y_{N}-y_{C_{i}}\right)}^{2}}$$

**Figure 2:**
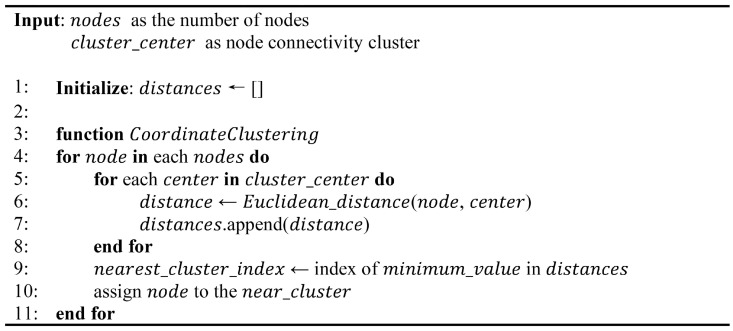
Pseudocode for node granting and optimal K selection: Coordinate clustering.

Equations (2) and (3) ensure that the cluster centers ($x^{\prime }c_{i}$,$y^{\prime }c_{i}$) consistently improve to accurately reflect the spatial distribution of nodes within each cluster $C_{i}$ after the initial assignment, as represented by $\left|N_{C_{i}}\right|$. Additionally, our method employs the elbow method and Silhouette Score in Equation (4) to determine the optimal number of clusters *k*. The elbow method identifies the ideal number of node clusters by detecting the point where the reduction rate in the within-cluster sum of squares (WCSS) significantly changes. Similarly, the Silhouette Score measures the compactness and separation of clusters, with higher scores indicating more distinct clusters. The algorithm illustrated in Figure 2, outlines the procedures involved in the optimization process, including node assignment and optimal *k* selection.

**Figure 3:**
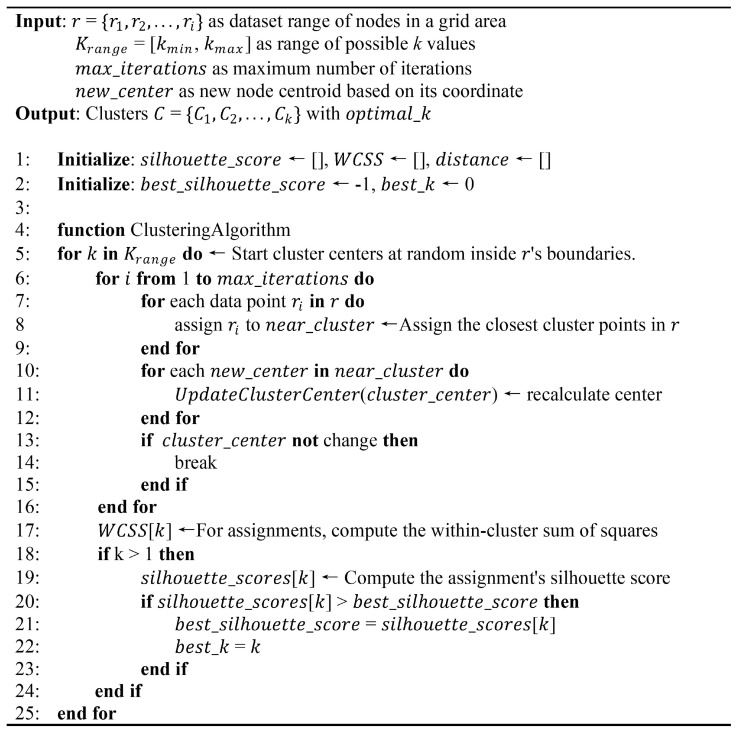
Pseudocode for node granting and optimal K selection: Clustering algorithm.

The algorithm begins by randomly initializing cluster centers and assigning nodes to the nearest cluster based on Euclidean distance. For each *k* value in the specified range, the algorithm computes the WCSS using Equation (4), with *x* and *y* coordinate points and ($\mu_{i}$) centroids used to evaluate the clustering quality and iteratively update the cluster centers. Consequently, the algorithm shown in Figure 3, determines the optimal number of clusters for the final K-means clustering phase.

(2) $$x^{\prime }c_{i}=\frac{1}{\left|N_{C_{i}}\right|}\sum_{N\in N_{C_{i}}}x_{N}$$

(3) $$y^{\prime }c_{i}=\frac{1}{\left|N_{C_{i}}\right|}\sum_{N\in N_{C_{i}}}y_{N}$$

## 5. Scheduling of TSCH

The MSF organizes these slot frames into TX and RX cells for each node, considering communication requirements and network grouping; parent nodes in the MSF coordinate slot frame allocations, while child nodes receive these allocations to enable communication. Typically, nodes send packets to a parent node, but if the parent node is unavailable, a child node can act as a hop node to forward packets to the sink node. Slot and channel availability are considered during cell allocation; hop nodes select the slot offset, channel, and TX and RX cells for communication between child nodes. Simultaneously, the root or sink node applies the same algorithm to determine the optimal cell configuration. Custom cell allocation involves determining the specific cell configuration allocated exclusively to each child node. The hop node assigns a slot offset to each child node based on the number of allocated cells ($N_{\mathrm{cells}}$), ensuring that each cell has a distinct offset. The root or sink node employs the same algorithm to determine exclusive cell allocations for each child node through the predetermined cluster head.

**Figure 4:**
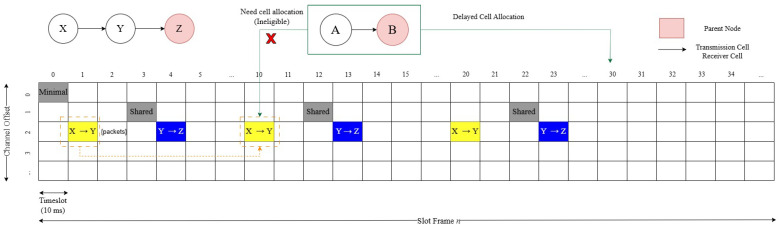
TSCH scheduling based on period traffic.

In the slot frame, which is a crucial component of the data transmission process, multiple nodes (such as Node A and Node B) may attempt to occupy the same cell allocation. This inherent allocation process can lead to conflicts and interference, reducing the packet delivery ratio and increasing latency. In addition, the latency can increase significantly for every 0.1 second delay per packet. In the absence of node clustering, nodes compete for optimal cell allocation within the slot frame. As network density increases, this competition becomes more intense, complicating the allocation process and reducing efficiency. The high volume of nodes vying for limited slots can disrupt allocation and lead to significant delays in data delivery. Additional cell allocations may be required to manage traffic spikes and meet strict latency requirements in order to ensure smooth and timely communication, as illustrated in Figure 4.

**Figure 5:**
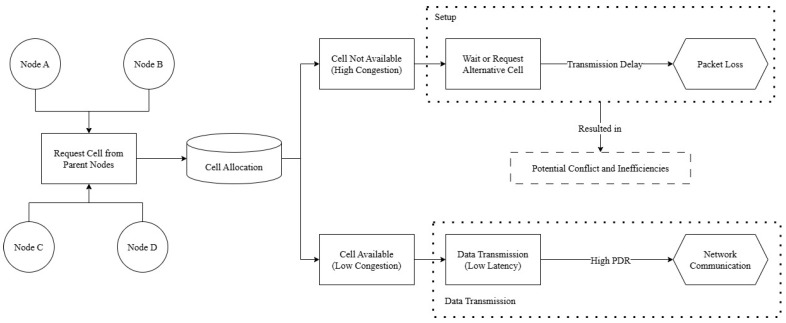
As-is diagram of default cell allocation in TSCH.

As shown in Figure 5, the cell allocation process at each node begins by connecting multiple nodes back to the central point, which is the parent node, in order to obtain available cells. If cell availability is low due to high density, nodes must wait or request an alternative cell, leading to delays and packet loss. When cells are available, data can be sent with low latency; however, conflicts and inefficiency can still arise, especially when node density is low.

**Figure 6:**
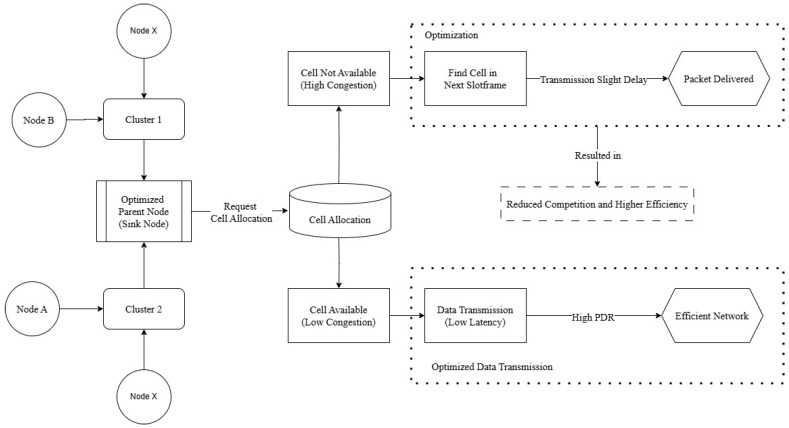
To-be diagram of cell allocation through clustering approach.

In contrast, Figure 6 illustrates an advanced method that utilizes K-means algorithm-based clustering to partition nodes based on their spatial positions and designates an optimized parent node, called the sink node. The resulting clusters inform a more efficient cell allocation process by assessing the cell requirements for each allocated frame slot. This method ensures that the allocation can continue seamlessly to the subsequent frame slot with minimal delay, even in the absence of an available cell. This approach effectively reduces node competition to maintain a high packet delivery ratio.

## 6. Cell Allocation Implication of Nodes

**Figure 7:**
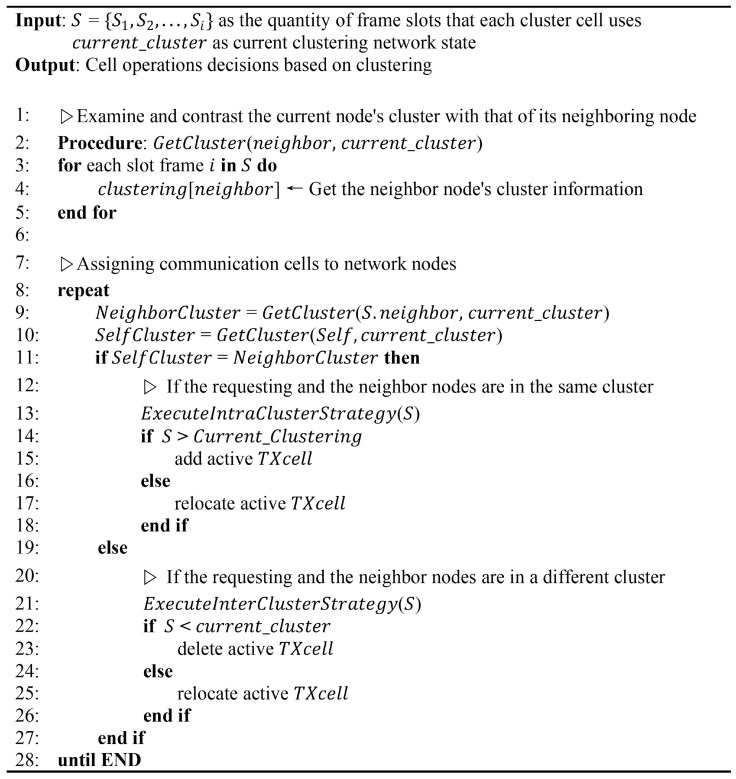
Pseudocode for enhanced TSCH cell allocation based on clustering.

As illustrated by the algorithm in Figure 7, the improved cell allocation method optimizes communication cell allocation in the TSCH network. The algorithm begins with inputs such as *S*, representing the number of slot frames used by cells in a cluster, and current_cluster, indicating the network’s current clustering status. It then compares a node’s current cluster association with its neighbors using GetCluster and evaluates each slot frame in *S* to collect cluster details of neighboring nodes by calculating the number of slot frames utilized during a given period for each cell involved. Each randomly determined node location result is selected by looking at the distance between the modes to be determined in the inter-cluster or intra-cluster category, which is then used as a reference when determining the sink node. The main execution phase continuously processes cell assignments until termination conditions are met, with the node acting as a child node performing the scheduling function through 6p transactions. This phase aims to divide the determination of each cell for the allocation of frame slots that have been determined based on the shared cells available for each frame slot used, in which case the cell is updated.

Information such as time slots and available channels is submitted to each clustered cell list. If the current node and its neighbors belong to the same cluster (as identified by GetCluster), then the intra-cluster strategy focuses on nearby nodes. Cluster formation enhances communication efficiency and improves time slot utilization. During this phase, active TX cells are added when the number of used slot frames exceeds the cluster capacity. When demand decreases, TX cells are reallocated to areas with higher user density, thereby optimizing resource usage and increasing throughput.

An inter-cluster strategy is proposed for nodes in different clusters using gateway or router nodes to facilitate communication between clusters, allowing the network to expand and adapt without disrupting operations. To prevent resource wastage, active TX cells are removed if fewer slot frames are available or are relocate to meet inter-cluster needs. Effective node clustering improves packet transmission efficiency by assigning specific cells based on a predetermined schedule. We consolidate resources by removing unnecessary cells, which reduces packet queuing and enables efficient communication between parent and child nodes. The 6P transaction processing minimizes signaling overhead and the nodes intelligently control cell usage, reducing 6P requests and acknowledgments while maintaining reliable connections.

**Figure 8:**
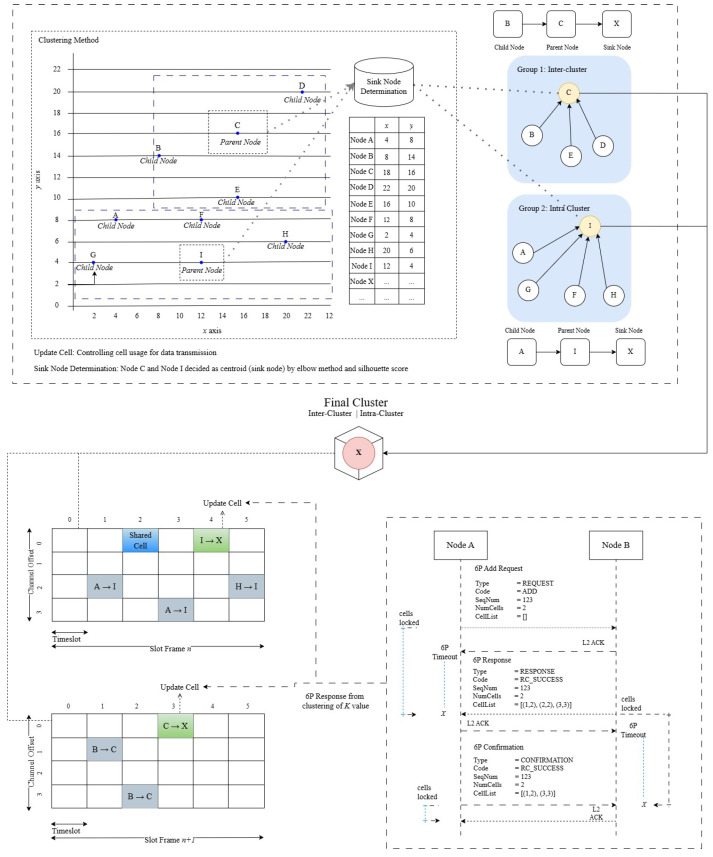
Scheme of the proposed clustering for cell allocation.

The proposed scheme, depicted in Figure 8, introduces innovative clustering-based cell allocation and dynamic reallocation strategies that enhance slot frame utilization and reduce signaling overhead, significantly improving network performance, as previously discussed regarding the K-means implementation. The K-means clustering process here has a complexity of approximately O(n×k×i), where *n* is the number of nodes, *k* is the number of clusters, and *i* is the number of iterations until convergence is achieved. The cell allocation process in the slot frames involves evaluating and assigning cells to each node in the cluster, with an estimated complexity around O(s×n), where *s* is the number of slot frames and *n* is the number of nodes. The optimized 6P transaction algorithm also considers cell usage based on dynamic demand, with the complexity being generally linear with the number of nodes.

## 7. Performance Evaluation

This section explains the simulators and parameters used in the experiments and our methods for comparing the routing protocols. It also explains how each RPL affects packet data transmission and how the node coordinate clustering results affect the tested performance metrics, including the packet delivery ratio, throughput, latency, and joining time of every node.

### Experimental Setup

To evaluate the effectiveness of our proposed K-means clustering approach, we compared it with other clustering methods, including GRID-based K-means (GBK) [13], ECRP clustering [17], and the default MSF [27]. All procedures were implemented on the same network layer in TSCH using the standard 6TiSCH simulator, with each node having a battery level of 2821.5 mAh on the same minimal scheduling function. The evaluation involved randomly placing nodes in an area for four scenarios involving 100, 150, 200, and 300 nodes, with each node positioned at specific coordinates. The number of nodes was chosen based on related works to ensure consistency. The optimal value for each parameter *k* was determined using the Silhouette Score and elbow method. Within the controlled environment of the 6TiSCH simulator, we carefully assessed the performance using the RPL. The assessment process included uniformly adjusting the packet transmission intervals up to 0.1 percent. Each network node produced 120 packets with 80 bytes of data, leading to a standardized transmission packet size of 1016 bits. This experiment was conducted using two different routing protocols. The first protocol was OF0 [32], which prioritizes paths with the highest packet delivery ratio among potential next-hop neighbors on the route to the root or parent node. The second routing protocol was MRHOF [33], which integrates metrics such as rank, which indicates link quality, and incorporates hysteresis to prevent excessive path changes, thereby minimizing latency and overhead.

(4) $$\;\mathrm{Throughput}\;\left(\mathrm{kbps}\right)=\frac{\;\mathrm{Received}\;\mathrm{packet}\;\times \;\mathrm{Packet}\;\mathrm{size}\;}{\;\mathrm{Simulation}\;\mathrm{time}\;\mathrm{allocation}\;}$$

Our work illustrates how a node-based clustering strategy can improve the 6TiSCH network’s scalability and performance. Network performance was measured using reliability indicators such as PDR, joining time, end-to-end latency, and throughput. PDR measures the percentage of successfully delivered packets among all transmitted packets to determine how reliable the network is at providing data. Measuring the time required for a packet to travel from its source to its destination provides the end-to-end latency, indicating how responsive the network is. The joining time indicates how quickly a new node integrates into the network. Throughput, as assessed by Equation (4), measures the data rate successfully sent from one node to another within a certain period or the number of packets sent from each source node to the destination node while considering the packet size, and is measured in kilobits per second (kbps). Table 1 displays the parameter simulations along with our suggested approach.

## 8. Results

### 8.1. Objective Function Zero (OF0) Routing Protocol

**Figure 9:**
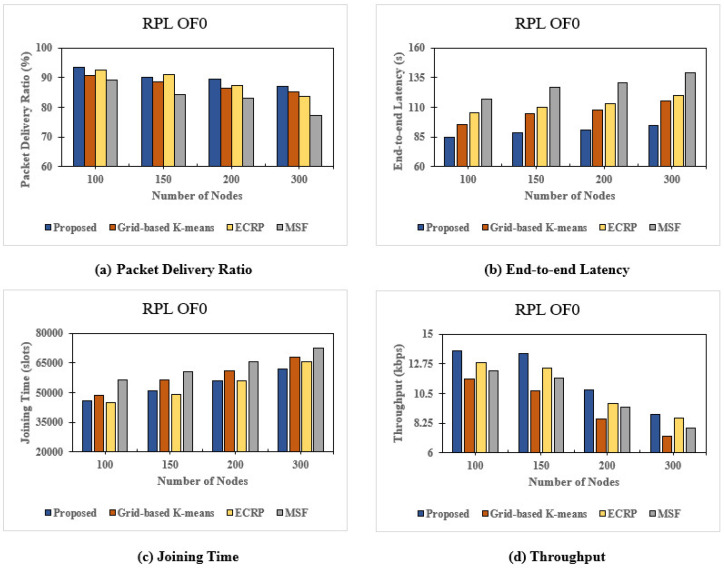
Graphs showing results for the Objective Function Zero (OF0) routing protocol: (**a**) Packet Delivery Ratio (PDR), (**b**) end-to-end latency, (**c**) joining time, and (**d**) throughput.

We initially conducted a performance test using the Objective Function Zero (OF0) routing protocol. This test compared our proposed clustering method with other methods and the MSF, as shown in Figure 9. Our method maintained a consistent delivery ratio across varying node counts. However, ECRP clustering slightly surpassed it at 150 nodes due to its cluster head selection based on node energy, as this value may be slightly higher due to the previously described clustering process affecting the scheduling process for each frame slot. The minimum hop count process in OF0 also affects inter-cluster communication. Despite this, our method’s iterative clustering and scheduling processes effectively predicted packet arrival, outperforming the grid-based clustering method. Overall, our clustering process yielded positive results. Figure 9 also demonstrates that the end-to-end latency with the OF0 routing protocol remained under 100 s even with 300 nodes, which can be attributed to the effective distribution of slot frames and time slots. The end-to-end latency for clustering on cell allocation also showed critical improvements compared to grid-based K-means. Noteworthy here is that slot frame distribution slightly affects latency, as the node coordinates used in slot frame distribution are divided appropriately for slot frames and individual time slots.

Figure 9 highlights the consistent joining time across different node counts, with K-means clustering reducing the time needed for new nodes to receive advertisements from the sink node. Energy efficiency in clustering remains high, mainly due to residual energy considerations for each node group. This allows our method to optimize how the sink node manages packet delivery for reserved nodes. The energy efficiency of clustering tends to be high in the following moments, as it heavily relies on the residual energy of each group of nodes; thus, our proposed method could determine how the sink node might accept and deliver some packets for reserved nodes. Figure 9 shows favorable throughput results, with each packet’s transmission speed enhanced by OF0’s low-overhead path selection. We believe that the throughput value will still be high through packet sending, as it might barge the transmission cells. Energy efficiency clustering reduces the throughput margin to 8.89 kbps at 300 nodes, yet overall throughput stays high due to effective packet transmission. In summary, our proposed method excels in packet delivery ratio and throughput, showing an 8% increase in delivery ratio, a 30.01% reduction in latency, a 15.95% decrease in joining time, and a 13.82% increase in throughput compared to the MSF under the OF0 routing protocol.

### 8.2. Minimum Rank with Hysteresis Routing Protocol

Our next experiment utilized the Minimum Rank with Hysteresis Objective Function (MRHOF) routing protocol. We used the same parameters as for OF0 and comparing the results with the default MSF. Our proposed clustering method directly allocates cells to each slot frame, facilitating packet retransmission to the destination node, especially in high-density clusters. This method achieved slightly better PDR percentage results than other clustering techniques; unlike OF0, it also supports multi-path routing. Clustering improves PDR by allocating cells directly. The clustering result improves the PDR value by directly determining the cells to be used. The highest PDR, observed with 100 nodes, reached 98.15%. This decreased to 93.35% with 300 nodes, reflecting the increased traffic load on network resources. The end-to-end latency performance metric remained stable despite a slight increase compared to grid-based K-means, reaching 112.91 s for 300 nodes due to fewer retransmissions.

**Figure 10:**
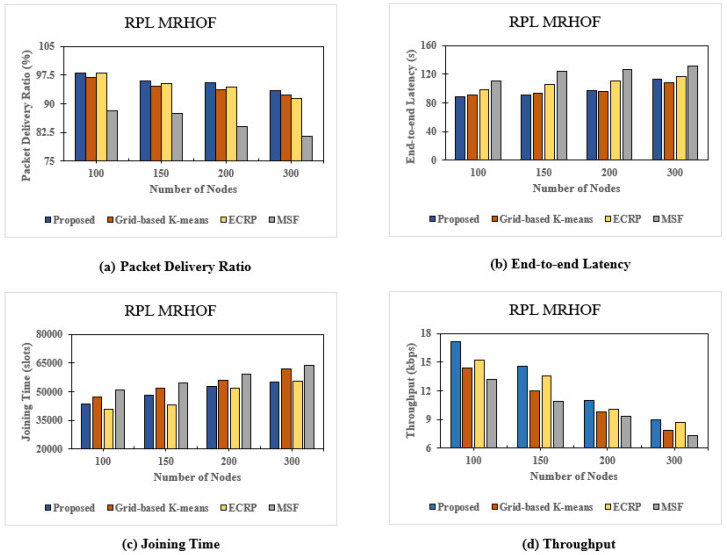
Graph results of the Minimum Rank with Hysteresis (MRHOF) routing protocol: (**a**) Packet Delivery Ratio (PDR), (**b**) end-to-end Latency, (**c**) joining time, and (**d**) throughput.

The joining time was consistent with other K-means clustering methods, achieving 55,219 slots compared to 55,394 slots for ECRP clustering. Congestion at the cluster head delays the transmission queue when transmitting to other slot frames, as depicted in Figure 8. Increased data traffic or competition for cluster resources likely caused congestion between cluster heads, delaying the transmission queue. Figure 10 also shows the throughput values for the data transmission speed, which differ from the OF0 RPL. Throughput, which is closely related to PDR, decreased from 17.12 kbps to 9 kbps as the number of nodes increased from 100 to 300, likely due to higher message transmission during the 6p transaction in grid-based K-means; the throughput decreases linearly as the number of nodes increases. Figure 10 highlights an anomaly in which fewer nodes encountered difficulties due to clustering on low nodes in top-6 protocol cell allocation, resulting in performance similar to MSF without clustering. Compared to MSF, our method showed a 12.34% increase in average PDR, a 21.06% reduction in latency, a 12.68% decrease in joining time, and a 25.97% increase in throughput speed.

## 9. Conclusions

This paper demonstrates the application of node clustering results using the K-means method for cell allocation in the 6TiSCH network utilizing node position data and segmenting based on OF0 and MRHOF, which influences routing paths and network organization. Our method involves positioning nodes, clustering node coordinates, determining cluster centers, designating centroids as cluster heads, and associating other points with the nearest centers without repeated iterations. The goal is to minimize packet hops in packet routing and reduce neighbor discovery, improving performance efficiency and network traffic stability in the 6TiSCH network. RX and TX cells are essential for creating an efficient slot schedule in a slot frame, while the system allocates each node a time slot for receiving RX cells and transmitting TX cells. Performance can vary while adhering to the standard scheduling function in the 6TiSCH network. Utilizing node position patterns allows K-means clustering to optimize resource allocation in order to manage resources based on cluster density. The clustering results determine the number of slots arranged in a time frame, while cell operations are adjusted through 6p transactions using K-means. The algorithm forms clusters of nodes based on proximity, optimizing specific cell allocation. In high-density scenarios, it adds dedicated cells to accommodate increased demand and removes redundant cells in low-density situations. At the same time, the strategy prioritizes allocating unicast cells immediately after the receiving cell, which is intended to minimize latency. Our performance results regarding packet delivery ratio, packet latency, node joining time, throughput, and data transmission success are promising. Evaluations across different node scenarios and comparisons with other clustering methods show significant improvements in average performance metrics. Under the Objective Function Zero (OF0) routing protocol, we achieved a 30.01% decrease in latency, a 15.95% reduction in joining time, an 8% increase in packet delivery ratio, and a 13.82% increase in throughput. Using the Minimum Rank with Hysteresis Objective Function (MRHOF) routing protocol, the average packet delivery ratio increased by 12.34%, latency decreased by 21.06%, joining time decreased by 12.68%, and throughput speed increased by 25.97%. The number of nodes in the network was assumed to be constant in order to maintain the focus on optimizing cell allocation and clustering techniques. In real-world scenarios, node loss from hardware failure, signal weakening, or environmental shadowing can alter network topology. Future research could incorporate models that address dynamic conditions and varying network sizes, or explore hybrid systems that combine multiple clustering techniques involving multi-objective allocation. These advancements could enhance algorithm performance by achieving energy savings through optimal time slot selection and improving scalability for faster response times with the alignment of other objective functions.

## Figures and Tables

**Table 1:** Simulation parameters.

Parameter	Value
Simulation area (grid size)	2 km × 2 km
Simulation platform	6TiSCH
Battery level	2821.5 mAh
Number of nodes	100, 150, 200, 300
RPL extensions	Unicast
RPL DAO interval	60 s
RPL objective function	OF0, MRHOF
Traffic period	Periodic
Node distribution	Random
TSCH number of channels	16
TSCH TX queue size	12 frames
TSCH timeslot length	10 ms
K maximum value	10
K-means maximum iterations	120
K-means features	3
Slot frame per run	4800
Packet size	1016
Silhouette score	−1
Silhouette cluster	3

## Data Availability

Data are contained within the article.
